# Editorial for the Special Issue on Recent Advances in Micro/Nano-Fabrication

**DOI:** 10.3390/mi15121485

**Published:** 2024-12-11

**Authors:** Yao Liu, Jinjie Zhou

**Affiliations:** School of Mechanical Engineering, North University of China, Taiyuan 030051, China

This Special Issue of *Micromachines*, named “Recent Advances in Micro/Nano-Fabrication”, comprehensively dedicates the latest research on micro/nano-fabrication in various fields. This Special Issue constitutes a synthesis of sixteen papers that investigate the most recent advancements and challenges in micro- and nanoscale fabrication processes. Micro/nanostructures have gained significant attention and wide applications in photocatalysis, coated fabrics, microchips, and sensors [1]. With the continuous increase in modern production demands, the capability to prepare microscopic patterns and structures is crucial for the advancement of many technologies and devices [2]. Removing micro- and nanoscale materials to achieve ultrahigh precision is also challenging. Surface micro/nanostructures generally possess unique textural structures [3] and exhibit certain periodicity [4], which makes them the preferred choice for achieving important performance characteristics in various devices. In the past decade, with technological advancements and the emergence of cost-effective manufacturing methods, the successful implementation of engineered surfaces with self-cleaning properties in industrial applications has been achieved [5]. The pursuit of smaller, multifunctional microdevices and integrated microsystems with superior electrical and mechanical performances stimulated micro/nano-fabrication technology breakthroughs [6]. By advancing the manufacturing technology of micro/nanostructures and their interfaces with industrial applications, this Special Issue presents the latest research progress in the micro/nano-fabrication process. The commonly used micro/nano-fabrication methods include electrochemical machining (ECM) [7], electrical discharge machining (EDM) [8], conventional mechanical machining [9], and laser beam machining (LBM) [10]. For example, ECM and EDM have been applied to fabricate the brain electrode in a microscale to reduce the damage to the tissue and nervous system during the invasion process [11]. Ultrasonic machining is also used to generate structure surfaces by using high-frequency vibration [12]. The microstructure was fabricated by the laser or chemical machining for the coating to find the super hydrophilic or superhydrophobic surface [13]. Laser transfer printing technology was used to make the wire in the photovoltaic panel with a thickness of less than 40 µm for high photoelectric conversion efficiency [14]. The micro/nano-fabrication process tackles challenges like high power consumption, low integration, and poor performance. Despite complexities and high costs, micro/nano-fabrication facilitates high-performance products and structures, which expand into ultra-large-scale integrated circuit manufacturing, nanoscale electronics, optoelectronics, high-density magnetic storage devices, microelectromechanical systems, biochips, and nanotechnology. Those applications offer even greater development opportunities for micro/nanofabrication.

The following contributions in this Special Issue cover a wide range of applications and processing techniques, providing valuable insights for the fabrication of various complex material surfaces:

A review article by Denys et al. [15] compared classical and recently developed scanning strategies using laser surface texturing (LST). Plasma and particle shielding effects and heat accumulation were described as fundamental physical limitations of short and ultrashort LSTs. Several methods of laser beam shifting, including the scanning strategy, straight shadow lines, path archiving, cross-coupled texture, and Lissajous or Hilbert curve filling, were discussed. The final discussion shows that a combination of several techniques, such as multi-beam processing, asynchronously shifted LST strategies, and Laser-Induced Periodic Surface Structure formation, can provide a way to achieve higher processing rates in LST. The subsequent phase in attaining elevated standards of Industry 4.0 may be the implementation of dynamic non-contact infrared temperature control in conjunction with high-speed scanning technology in laser surface treatment.

The latest abrasive processing techniques for SiC ceramic composites were reviewed by Zhang et al. [16]. Both conventional grinding (CG) and non-conventional grinding (non-CG) techniques were introduced and compared the advantages and disadvantages. Subsequently, four simulation methods, namely the finite element method (FEM), smooth particle hydrodynamics (SPH), molecular dynamics (MD) analysis, and discrete element method (DEM) analysis, are presented briefly, with an overview of the fundamental theory, the current status of the research, and the scope of application. It is proposed that the application of multiscale simulation methods for the study of ceramic matrix composite (CMC) grinding is a promising method. Furthermore, the particular structural and physical characteristics of CMCs are examined, and the findings indicate that reinforcing fibers not only address the inherent deficiencies in CMC materials but also alter the removal mechanism. Ultimately, it is recommended that the utilization of novel technologies to enhance the quality of grinding, comprehend the influence of fiber orientation and distribution on the removal mechanism and removal mode of the material, and develop a suitable machining model are crucial avenues of future research.

In a review of femtosecond laser processing on silicon carbide, Wang et al. [17] provided a comprehensive analysis of the machining techniques and applications of femtosecond laser on SiC. Various femtosecond laser hybrid fabrication methods and the utilization of femtosecond laser machining SiC structures in microelectromechanical systems (MEMS) were discussed. The femtosecond laser can machine pits, corrugated structures, nanoscale structures, grooves, and large surface areas directly on SiC. Additionally, femtosecond lasers can be used in conjunction with other processing techniques to machine SiC, such as water jet processing, underwater processing, and chemical selective etching. The advantages of femtosecond laser machining SiC for sensors, microcavities, and micromechanical systems were also presented. The femtosecond laser hybrid fabrication techniques address the limitations of high hardness, excellent thermal conductivity, and the chemical inertness of SiC semiconductors, which promote femtosecond laser machining technique development and application. This provides an important direction for exploring advances in femtosecond laser micro- and nanofabrication, with the goal of diversifying the processes used to fabricate SiC devices.

Peng et al. [18] developed a photolithography model based on optical imaging and photochemical reaction profiles. The study proposed a sub-domain division method with statistical principles to enhance the efficiency and precision of 3D Optical Proximity Correction (3D OPC). This method was then applied to fabricate high-fidelity micro-Fresnel lenses. The proposed 3D OPC method for fabricating Fresnel lenses demonstrated a significant reduction of 79.98% in profile error. Additionally, the average peak signal-to-noise ratio (PSNR) of the images utilized in the imaging system exhibited an 18.92% enhancement, while the average contrast of the images showed a 36% improvement. Those outcomes validated the reliability of the algorithm. Furthermore, the potential of this technique in future work to use 3D OPC algorithms to optimize achromatic lenses to improve the PSNR and the contrast of imaging was discussed.

Kim et al. [19] employed a selective laser etching (SLE) technique to fabricate a connection hole on the glass-mediated layer (TGV). The objective was to identify the most effective etchant to improve the etching rate. Four different etchants (HF, KOH, NaOH, and NH_4_F) were compared. The results demonstrated that the most effective etchant was NH_4_F. The TGV could be generated within 3 h through etching with the 8 M NH_4_F solution at 85 °C. The HF, NH_4_F, NaOH, and KOH solutions generated cone angles of 1–53°, 47–60°, 53–62°, 53–62°, and 58–66°, respectively. The optimal process parameters were found to enhance the productivity of TGVs in glass-mediated layers.

A study on micromirror arrays with tunable reflective properties based on different microstructures and their applications was conducted by Cao et al. [20]. The results demonstrated that microstructures prepared using 3D lithography can be used to tune the reflective properties without the need for an additional power supply. The microstructures were also applied to the projection display segment, which showed that the maximum surface gain of the concave–convex aspheric micromirror arrays (MMA) was up to 2.66. The maximum surface gain of less than 2.72 had a small color difference, minimal geometric distortion, and high-gain reflective properties, which provided enhanced performance and reduced processing costs compared to existing mainstream optical screens. This study addressed the limitation of the modulation of reflective property adjustment due to the complex structure and preparation process in power systems, which provided new opportunities for the development of advanced reflective optical surfaces.

Man et al. [21] fabricated parallel interconnects using a digital design and investigated the effect of Triton X-100 dispersant on the rheology of La_0.6_Sr_0.2_Fe_0.8_Co_0.2_O_3_-δ (LSCF) slurries. This was achieved by using a direct-write micromanipulation technique to fabricate micro-single-compartment SOFCs (u-SC-SOFCs). The results indicated that the optimal concentration of Triton X-100 for various slurries was approximately 0.2–0.4% of the LSCF solid in weight. In detail, the slurry composition of 50% solids, 12% binder, and 0.2% dispersant was identified as the optimal formulation for SOF fabrication via the direct-write method. This study established a foundation for the development of optimal process parameters and sintering cycles for the deposition of high-fidelity cathode electrodes for miniature single-compartment solid oxide fuel cells for green energy.

Li et al. [22] employed dispersion polymerization to synthesize micro/nanoscale polystyrene (PS) spheres, which were subsequently deposited onto a silicon substrate via a floating assembly method to form a long-range monolayer. Sub-wavelength structures were subsequently manufactured using dry etching techniques. The polystyrene spheres with diameters ranging from 500 nm to 5.6 µm were produced by making adjustments to the polyvinylpyrrolidone (PVP) stabilizer and variations in component concentration. The optical limitations inherent in colloidal lithography in the conventional lithography process were resolved.

In a study by Li et al. [23], a superhydrophobic substrate, developed via a novel method using a bottom-up scheme, was fabricated using stoichiometric-grade thiol-ene (OSTE) micro-mushroom forests. In a relatively short time period, a large area of OSTE micro-mushroom forests was fabricated, which exhibited excellent superhydrophobicity and low adhesion properties. Those properties were evidenced by a contact angle of 152.9 ± 0.2°, a sliding angle of 4.1°, and a contact angle hysteresis of less than 0.5°. The deficiencies of the traditional fabrication methods for superhydrophobic surfaces were solved in this study. Those properties indicated that this substrate has great potential for superhydrophobic applications.

Kim et al. [24] employed the evolutionary, finite element, machine learning, and deep learning methods to model the photoresist flow process (RFP) under various reflow conditions. Additionally, the self-consistent field theory (SCFT) was utilized to describe the self-assembly of a cylindrical block copolymer (BCP), which was confined in the pre-patterning of the photoresist reflow process (RFP) to produce small contact hole (C/H) sizes. The study established a foundation for shrinkage modeling and the generation of smaller contact holes, which enhanced the photoresist reflow process (RFP) with random contact holes (C/Hs).

The nanosecond laser direct-write ablation technique was employed to create a micro- and nano-textured surface on a high-frequency electrosurgical knife [25]. The surface was designed to mimic the structure found on the leaves of Setaria viridis. Additionally, the TiO_2_ coating was deposited on the knife surface through magnetron sputtering. Then, the plasma-induced hydrophobic modification and octadecyl trichlorosilane (OTS) treatments were conducted to enhance the hydrophilic nature of the silicone oil. These can construct a self-lubricating and anti-stick surface. The study successfully reduced the tendency of human tissues to adhere to electrosurgical knives, which can reduce the risk of surgical complexity and increased medical complications.

Luo et al. [26] investigated the mechanism of the in situ laser-enhanced ultra-precision cutting machinability of ZnS. The physical properties of ZnS were characterized by high-temperature nanoindentation experiments. The material removal mechanism of ZnS during in situ laser-assisted cutting was also investigated. The results revealed that the initiation depth of the groove damage produced by in situ laser-assisted cutting increased by 57.99%. The formation of micrometer-scale pits was effectively mitigated through the use of in situ laser-assisted cutting. Following the planning of cutting experiments, it was successfully demonstrated that a smooth and uniform surface with a Sa of 3.607 nm was achieved at a laser power of 20 W, which was 73.58% higher than that achieved through normal cutting. The study provided a promising method for ultra-precision cutting of ZnS.

Singh et al. [27] developed CAM software (version 1) for aspherical surface machining by integrating the measurement technology with an optimized material removal mechanism in grinding and polishing. Computer numerical control (CNC) technology, computer technology, and data analysis were also combined. The software employs compensating correction algorithms to process error data and generate numerical control programs for machining. Thie study addressed the existing manufacturing challenges and improved the performance of the optical system.

Shi et al. [28] developed a normal displacement model based on the Hertz contact theory. A normal displacement compensation method was subsequently proposed based on the decoupled polishing system. The experimental results demonstrated that compared to no displacement compensation, the utilization of the normal displacement compensation during the polishing process resulted in a notable reduction in the surface roughness from 0.4 µm to 0.21 µm. Additionally, the unevenness coefficient of surface roughness exhibited a considerable decline from 112.5% to 19%. This study solved the polishing tool location error caused by the contact deformation of the tool, abrasive, and workpiece.

Duan et al. [29] fabricated layered micro/nanostructures, which comprise periodic microstructures, laser-induced periodic surface junctions (LIPSSs), and nanoparticles, through femtosecond laser processing (LP). Furthermore, a hydrophobic layer was formed on the micro/nanostructures through perfluorosilane modification (PM). The experiments were conducted under the optimized parameters, and the results demonstrated that the average reflectivity on the silicon surface in the visible light band was reduced to 3.0%. Additionally, the surface exhibited a large contact angle of 172.3 ± 0.8° and a low sliding angle of 4.2 ± 1.4°. This approach has potential applications in several fields, including optics, detectors, and photovoltaics.

In the last contribution, Liu et al. [30] conducted sandblasting experiments to investigate the effects of process parameters (air pressure, lift-off height, abrasive volume, and abrasive type) on the processing time, surface roughness, and mechanical properties of wire component. The goal was to reveal the different cutting mechanisms between the SAC solder and 304 V wire. The results demonstrated that resin abrasives can effectively remove SAC burrs while maintaining the integrity of 304 V due to the proper hardness and young modulus. The processing time decreased with increasing air pressure, while the surface roughness increased with increasing abrasive volume. Additionally, sandblasting led to a decrease in the yield strength of the wire. The tensile strength was influenced by Young’s modulus and the hardness of the abrasive. The study provided a comprehensive understanding of sandblasting and the different cutting mechanisms.

In conclusion, this Special Issue of *Micromachines* offers a comprehensive and insightful overview of the latest developments in micro/nano-fabrication. The processing methods and combinations of techniques presented in these papers provide innovative ideas for the future micro/nano-fabrication of materials and promote the application of micro/nano-fabrication technologies in a wide range of fields. The findings of the above studies provide researchers with valuable insights that can be employed to further develop and enhance the utilization of micro/nano-fabrication techniques in electronic devices, semiconductors, and bio-devices. This will lead to improved product performance and value.
